# Transbronchial Lung Cryobiopsies, Transbronchial Forceps Lung Biopsies, and Surgical Lung Biopsies in Mechanically Ventilated Patients with Acute Hypoxemic Respiratory Failure: A Retrospective Cohort Study

**DOI:** 10.1177/08850666241247145

**Published:** 2024-04-22

**Authors:** Qi Li, Dominique Lafrance, Moishe Liberman, Charles Leduc, Emmanuel Charbonney, Polina Titova, Hélène Manganas, Michaël Chassé

**Affiliations:** 1 Department of Medicine, 12368 Université de Montréal, Montréal, Canada; 2 Division of Intensive Care, 177460 Centre Hospitalier de l’Université de Montréal, Canada; 3 Division of Thoracic Surgery, Endoscopic Tracheo-bronchial and Oesophageal Centre, 12368 Centre Hospitalier de l’Université de Montréal, Canada; 4 Centre de recherche du Centre Hospitalier de l’Université de Montréal, Canada; 5 Department of Pathology, 25443 Centre Hospitalier de l’Université de Montréal, Canada; 6 Division of Pulmonology, 25443 Centre Hospitalier de l’Université de Montréal, Canada

**Keywords:** respiratory insufficiency, biopsy, ventilators, mechanical, bronchoscopy, thoracic surgery, video-assisted

## Abstract

**Importance:**

Lung biopsies are sometimes performed in mechanically ventilated patients with acute hypoxemic respiratory failure (AHRF) of unknown etiology to guide patient management. While surgical lung biopsies (SLB) offer high diagnostic rates, they may also cause significant complications. Transbronchial forceps lung biopsies (TBLB) are less invasive but often produce non-contributive specimens. Transbronchial lung cryobiopsies (TBLC) yield specimens of potentially better quality than TBLB, but due to their novel implementation in the intensive care unit (ICU), their accuracy and safety are still unclear.

**Objectives:**

Our main objective was to evaluate the risk of adverse events in patients with AHRF following the three biopsy techniques. Our secondary objectives were to assess the diagnostic yield and associated modifications of patient management of each technique.

**Design, Settings and Participants:**

We conducted a retrospective cohort study comparing TBLC, TBLB, and SLB in mechanically ventilated patients with AHRF.

**Main Outcomes and Measures:**

The primary outcome was the proportion of patients with at least one complication, and secondary outcomes included complication rates, diagnostic yields, treatment modifications, and mortality.

**Results:**

Of the 26 patients who underwent lung biopsies from 2018 to 2022, all TBLC and SLB patients and 60% of TBLB patients had at least one complication. TBLC patients had higher unadjusted numbers of total and severe complications, but also worse Sequential Organ Failure Assessment scores and P/F ratios. A total of 25 biopsies (25/26, 96%) provided histopathological diagnoses, 88% (22/25) of which contributed to patient management. ICU mortality was high for all modalities (63% for TBLC, 60% for TBLB and 50% for SLB).

**Conclusions and Relevance:**

All biopsy methods had high diagnostic yields and the great majority contributed to patient management; however, complication rates were elevated. Further research is needed to determine which patients may benefit from lung biopsies and to determine the best biopsy modality.

## Introduction

### Background/Rationale

In patients with acute hypoxemic respiratory failure (AHRF) of unknown etiology, empirical treatment with antimicrobials and steroids is often used, though the benefits of this approach are uncertain due to the unknown nature of the illness and the potential for side effects. A histopathological diagnosis obtained through a lung biopsy may be useful for guiding patient management and prognosis.^1–3^ Studies have shown that histological results that contribute to treatment modifications may be associated with improved survival.^4,5^

Several modalities exist for performing lung biopsies. Surgical lung biopsies (SLB) are often considered the “gold standard” method, with high rates of diagnosis and contribution to management (78%-80%).^6–9^ However, most of them require intra-hospital transport and access to the operating room and carry the risk of air leaks, bleeding, and variable incidence of post-operative complications.^10,11^ Video-assisted thoracic surgery (VATS) requires single-lung ventilation, which is often not tolerated in patients with severe baseline lung injury and hypoxemia. Transbronchial forceps lung biopsies (TBLB) can be performed at the bedside and have the potential of fewer complications compared to SLB, but produce smaller specimens often with artifacts^12,13^ thus have a lower diagnostic yield and contribution to patient management (41%-58%).^5,14–16^ Transbronchial lung cryobiopsies (TBLC) are an alternative method for diagnosing interstitial lung disease.^17^ TBLC specimens are larger and have less artifacts compared to TBLB^10,11^ with consequently better diagnostic rates (84%)^18–21^ and high concordance with SLB results.^17,19^ TBLC have recently been introduced for patients with AHRF of unknown etiology in the intensive care unit (ICU), with reported diagnostic yields similar to or higher than TBLB and SLB (72%-100%)^5,22–24^ and variable complications rates. However, the safety and accuracy of TBLC compared to SLB and TBLB is not yet clear, and the choice between biopsy modalities often relies on the individual experiences of clinicians.

### Objectives

The main objective of our study was to investigate the risk of adverse events after lung biopsy in patients with AHRF. Our secondary objectives were to investigate the diagnostic yield and the associated modification of patient management for the various biopsy techniques.

## Methods

### Study Design and Patient Selection

We conducted a retrospective cohort study of mechanically ventilated patients with AHRF who underwent TBLC, TBLB, or SLB procedures at the Centre Hospitalier de l'Université de Montréal (CHUM) between January 1, 2018 and February 1, 2022. The study participants were identified through the Center for Integration and Analysis of Medical Data (CITADEL) and additional searches were performed in the CHUM medical archives and the division of thoracic surgery's records of lung biopsies. We included patients over the age of 18 who had AHRF and pulmonary infiltrates of unknown etiology. We excluded patients who did not require mechanical ventilation or who were placed on mechanical ventilation only after a lung biopsy was performed.

### Exposures: Lung Biopsy Procedures

TBLC and TBLB were performed at the bedside in the ICU. TBLC were performed by thoracic surgeons and TBLB were performed by interventional pulmonologists. A therapeutic flexible bronchoscope (working channel 2.8 mm; Olympus, Tokyo, Japan) was used for all TBLC procedures through the existing endotracheal tube with a bronchoscope swivel chimney adapter and the patient pre-oxygenated for 5 min with 100% FiO2. The cryoprobe utilized was either a non-disposable 1.9 mm flexible cryoprobe with N2O as the cryogen or a disposable 1.9 mm cryoprobe with CO2 as the cryogen (Erbe, Tuebingen, Germany). The probe was advanced in the airway and positioned in the targeted lobe based on the pre-procedural CT scan after discussing target location for biopsies with the multidisciplinary team (ICU, interventional pulmonology, radiology, thoracic surgery). Fluoroscopy was not used in our patients, and systematic insertion of bronchial blockers was not performed.

All SLB were performed in the operating room. Seven biopsies were performed by VATS and one by open thoracotomy in a patient under extracorporeal membrane oxygenation (ECMO).

Biopsy results and treatment modifications were discussed between the intensivist and the consulting pathologist, pulmonologist, and microbiologist.

### Outcomes and Data Collection

The aim of our study was to determine the proportion of patients who experienced one or more complications after lung biopsy and to assess the rates of each adverse event. We also sought to determine the histopathological diagnoses and their impact on patient management. To do this, we reviewed patients’ medical records and recorded the following adverse events: desaturation, pneumothorax, bronchopleural fistula, additional chest tube insertion post-biopsy, bleeding, hemodynamic instability, and death caused by biopsy. Bronchopleural fistula was defined as the presence of air bubbling in the chest drainage system 24 h after the procedure or the insertion of an additional chest tube. We classified the severity of desaturation using a scale of three categories: none, mild (increase in FiO2 < 30% or decrease in PaO2 < 30 mm Hg), and severe (increase in FiO2 > 30%, decrease in PaO2 > 30 mm Hg, saturation less than 90% but under 100% FiO2). In line with the previous Nashville Consensus Statement on bleeding characterization in transbronchial lung biopsies, we classified the severity of bleeding using a scale of four categories: grade 1 (less than 1 min of suctioning or wedging of the bronchoscope resulting in spontaneous cessation of bleeding), grade 2 (suctioning more than 1 min, need for re-wedging of the bronchoscope or instillation of cold saline, vasoactive substances, or thrombogenic agents), grade 3 (balloon/bronchial blocker for less than 20 min or premature interruption of the procedure), and grade 4 (packed RBC transfusion, need for bronchial artery embolization or resuscitation).^25^ We recorded the total number of overall and severe complications and calculated the mean number of complications per patient. Adverse events defined as severe complications included severe desaturation, post-biopsy chest tube insertion, bronchopleural fistulas, severe bleeding, severe and refractory hemodynamic instability, and death caused by biopsy. In addition, we recorded patient characteristics such as age, sex, comorbidities, Sequential Organ Failure Assessment (SOFA) scores, baseline blood gas values and ventilator settings 12 h prior to lung biopsy, diagnostic workup, use of empirical antimicrobials and corticosteroids prior to the biopsy, histological diagnoses, treatment modifications, ICU and hospital mortality, duration of mechanical ventilation post-biopsy, and duration of hospital stay. A biopsy was considered having a positive diagnostic yield when there is the presence of alveolar tissue with a clearly diagnosed histologic pattern by the pathologist, or with an uncertain diagnosis that was later confirmed by the clinical evolution.

### Statistical Analysis

At baseline, we described the characteristics of all study participants using descriptive statistics. We recognize the limited number of patients and outcomes in our study and thus kept our analyses simple. To estimate the precision of our complication rates, we calculated 95% confidence intervals using the Clopper-Pearson method for proportions and a bootstrap approach with 10 000 iterations for continuous values. Given the small sample size and imbalances at baseline, we did not perform hypothesis testing. All statistical analyses were carried out using R version 4.1.2.

### Patient Privacy and Ethics

The study protocol is in accordance with the ethical standards of the institutional research committee—the Research and Ethics Board of the CHUM, and with the Helsinki Declaration of 1975. Approval for this project under the name “Transbronchial cryobiopsies, transbronchial forceps biopsies and SLB in patients with AHRF under mechanical ventilation: a retrospective cohort study” was obtained from the Research and Ethics Board on September 28, 2022 [22.134 (2023-10821)]. Individual informed consent for the usage of data for this project was waived in accordance with the institutional regulations for research on medical records of the Research and Ethics Board of the CHUM.

## Results

### Patient Characteristics

We included a total of 26 patients in the study, with 8 in the TBLC group, 10 in the TBLB group, and 8 in the SLB group. Table 1 presents the participants baseline characteristics and their comorbidities. Their median age was 60 years in the TBLB group, 48 years in the TBLC group and 46 years in the SLB group. Nine of the 10 patients (90%) in the TBLB group had undergone prior lung transplantation. All patients in the TBLB group were immunosuppressed, compared to three out of eight patients in the TBLC group and two out of eight patients in the SLB group. Of the total patient population, 76% (20 out of 26) were admitted for respiratory failure, while 15% (4 out of 26) were admitted for lung transplantation. Two other patients were initially admitted for hematuria and rectal bleeding, respectively, and subsequently developed respiratory failure. The median SOFA score was 12 for the TBLC group, 11 for the TBLB group, and 8.5 for the SLB group. Patients undergoing TBLC required higher FiO2 compared to the other two groups. The median partial pressure of oxygen in arterial blood (PaO2) to fraction of inspired oxygen (FiO2) ratio was 143 for the TBLC group, 204 for the TBLB group, and 190 for the SLB group. Two patients received ECMO prior to lung biopsy, one from the TBLC group, and one from the SLB group. The mean peak inspiratory pressure was 33 cm H2O for TBLC patients, 30 cm H2O for TBLB patients, and 28 cm H2O for SLB patients (see Supplement Table 1). Inhaled vasodilators were administered to two TBLC patients, 1 TBLB patient and 1 SLB patient. Biopsies were performed after a median of 7 days of mechanical ventilation. Prior to the biopsies, BAL cell counts and cytology were ordered in 35% (9 out of 26) of patients and transthoracic echocardiography in 54% (14 out of 26) (see Supplement Table 2). Empiric antimicrobials were administered in 96% of patients (25 out of 26) and corticosteroids in 50% (13 out of 26).

**Table 1.** Baseline Characteristics and Clinical Findings.

Baseline characteristic	TBLC n = 8	TBLB n = 10	SLB n = 8	Total n = 26
Age (median, IQR)	48 (41)	60 (10)	46 (37)	58 (31)
Age range (n, %)				
18-59 years	5 (63)	4 (40)	6 (75)	15 (58)
≥60 years	3 (38)	6 (60)	2 (25)	11 (42)
Reported administrative sex (n, %)				
Female	4 (50)	5 (50)	3 (38)	12 (46)
BMI (median, IQR)	26 (8)	23(6)	27 (9)	26 (9)
Respiratory comorbidities (n)				
Former lung transplant	0	9	0	9
Other (Includes COPD, ANCA-associated vasculitis, COVID, cystic fibrosis)	2	2	3	7
Immunosuppression (n, %)	3 (38)	10 (100)	2 (25)	15 (57)
Diagnosis at admission (n, %)				
Respiratory failure	6 (75)	6 (60)	8 (100)	20 (76)
New lung transplant	0	4 (40)	0	4 (15)
Other	2 (25)	0	1 (13)	3 (12)
SOFA score (median, IQR)	12.0 (4.3)	11.0 (3.75)	8.5 (7.5)	11 (5)
Days under mechanical ventilation before lung biopsy (median, IQR)	5.5 (7.3)	8.5 (25.5)	11.5 (15)	7 (16)
PaO_2_/FiO_2_^a^ (median, IQR)	143 (126)	204 (177)	190 (120)	188 (120)
ECMO (n, %)	1 (13)	0	1 (13)	2 (8)
Empiric treatments (n, %)				
Antibacterial, antiviral or antifungal	8 (100)	10 (100)	7 (88)	25 (96)
Corticosteroids	4 (50)	4 (40)	5 (63)	13 (50)

BMI, body mass index; SOFA, Sequential Organ Failure Assessment; ECMO, extracorporeal membrane oxygenation; SLB, surgical lung biopsies; TBLB, transbronchial forceps lung biopsies; TBLC, transbronchial lung cryobiopsies.

a FiO2 and PaO2 values from the last arterial blood gas drawn within 12 h of the biopsy.

### Complications

All patients in the TBLC or SLB groups experienced at least one complication, while six out of 10 patients in the TBLB group had at least one complication (see Table 2). Patients in the TBLC group had higher rates of severe complications (an average of 1.9 severe complications per patient for TBLC, 0.3 for TBLB, and 0.8 for SLB). Post-procedure pneumothorax occurred in three out of eight patients (38%, 95% CI 8-76) in the TBLC group, two out of 10 patients (20%, 95% CI 3-56) in the TBLB group, and three out of 8 patients (38%, 95% CI 8-76) in the SLB group. Grade 3-4 bleeding events occurred in 2 out of 8 patients (25%, 95% CI 3-65) in the TBLC group, 0 out of 10 patients (0%, 95% CI 0-31) in the TBLB group, and 2 out of 8 patients (25%, 95% CI 3-65) in the SLB group. Administration of therapeutic anticoagulation within 48 h of the biopsy occurred for three patients in the context of renal replacement therapy (Supplement Table 2). Among them, one patient underwent TBLC which was immediately followed by severe bleeding, and two patients underwent TBLB with no bleeding complications. The mean duration of mechanical ventilation was 17 days (95% CI 12-28) for the TBLC group, 37 days (95% CI 23-58) for the TBLB group, and 28 days (95% CI 17-37) for the SLB group. The mean ICU stay was 19 days (95% CI 13-31) for the TBLC group, 54 days (95% CI 30-100) for the TBLB group, and 36 days (95% CI 23-52) for the SLB group. ICU mortality was high in all groups, with 5 out of 8 patients (63%, 95% CI 24-91) in the TBLC group, 6 out of 10 patients (60%, 95% CI 26-88) in the TBLB group, and 4 out of 8 patients (50%, 95% CI 25, 92) in the SLB group dying. One death was directly attributed to the biopsy procedure and occurred in a patient from the TBLC group. This patient, who had severe obesity and newly discovered neoplasia of unknown origin, developed severe persistent hypoxemia immediately after the biopsy and the medical team and family decided to transition to palliative care.

**Table 2.** Post-Biopsy Complications and Evolution.

	TBLC n = 8		TBLB n = 10		SLB n = 8		Total n = 26	
	n (%)	95% CI	n (%)	95% CI	n (%)	95% CI	n (%)	95% CI
Proportion of patients having at least one complication	8 (100)	63, 100	6 (60)	26, 88	8 (100)	63, 100	22 (85)	65, 96
Desaturation^a^								
None^b^	1 (14)^a^	0, 53	9 (90)	80, 100	1 (14)^a^	0, 54	11 (46)^a^	29, 69
Mild^b^	3 (43)^a^	14, 82	0	0, 16	5 (71)^a^	57, 100	8 (33)^a^	17, 57
Severe^b^	3 (43)^a^	14, 82	1 (10)	0, 26	1 (14)^a^	0, 54	5 (21)^a^	4, 44
Pneumothorax	3 (38)	8, 76	2 (20)	3, 56	3 (38)	8, 76	8 (31)	14, 52
Additional post-biopsy chest tube	3 (38)	8, 76	1 (10)	0, 45	0	0, 37	4 (15)	4, 35
Bronchopleural fistula	3 (38)	8, 76	1 (10)	0, 45	2 (25)	32, 65	6 (23)	9, 44
Bleeding^c^								
Grade 1	4 (50)	16, 84	9 (90)	55, 100	6 (75)	35, 97	19 (73)	52, 88
Grade 2	2 (25)	3, 65	1 (10)	0, 45	0	0, 37	2 (8)	1, 25
Grade 3	0	0, 37	0	0, 31	0	0, 37	0	0, 13
Grade 4	2 (25)	3, 65	0	0, 31	2 (25)	3, 65	4 (15)	4, 35
Hemodynamic instability								
None	5 (63)	38, 96	8 (80)	44, 97	6 (75)	63, 100	19 (73)	62, 92
Start or increase of vasopressors	0	0, 33	2 (20)	3, 56	1 (13)	0, 48	3 (12)	0, 30
Severe and refractory	3 (38)	13, 71	0	0	1 (13)	0, 48	4 (15)	4, 34
Death caused by biopsy	1 (13)	0, 53	0	0, 31	0	16, 84	1 (4)	0, 31
ICU mortality	5 (63)	24, 91	6 (60)	26-88	4 (50)	25, 92	15 (57)	37, 77
Hospital mortality	5 (63)	24, 91	8 (80)	44-97	5 (63)	25-91	18 (69)	48, 86
	Mean (± SD)	95% CI	Mean (± SD)	95% CI	Mean (± SD)	95% CI	Mean (± SD)	95% CI
Length of mechanical ventilation (days)	17 (± 11)	12, 28	37 (± 28)	23, 58	28 (± 15)	17, 37	28 (± 22)	21, 38
Length of ICU stay (days)	19 (± 13)	13, 31	54 (± 54)	30, 100	36 (± 22)	23, 52	38 (± 38)	27, 58
Length of hospital stay (days)	33 (± 19)	21,45	75 (± 65)	43, 121	54 (± 37)	33, 81	56 (± 48)	41, 77

^a^ Exclusion of two patients under ECMO before, during, and after lung biopsies (1 patient in the TBLC group and 1 patient in the SLB group).

^b^ Grading of desaturation severity: none, mild (PaO2/FiO2 reduction of > 30), severe (saturation < 90% with 100% FiO2).

^c^ Grading of bleeding severity (Nashville Bleeding Scale): 1 (less than 1 min of suctioning or wedging of the bronchoscope resulting in spontaneous cessation of bleeding), 2 (suctioning more than 1 min, need for re-wedging of the bronchoscope or instillation of cold saline, vasoactive substances, or thrombogenic agents), 3 (balloon/bronchial blocker for less than 20 min or premature interruption of the procedure), and 4 (packed RBC transfusion, need for bronchial artery embolization or resuscitation).

SLB, surgical lung biopsies; TBLB, transbronchial forceps lung biopsies; TBLC, transbronchial lung cryobiopsies; ICU, intensive care unit; ECMO, extracorporeal membrane oxygenation.

### Histopathological Findings and Modification of Patient Management

A mean of four biopsies (2 to 5) from one lobe were performed in patients undergoing TBLC. A mean of seven biopsies (5 to 15) from one lobe were performed in patients undergoing TBLB. Two–three wedge specimens were collected from two to three lobes in patients undergoing SLB.

**Figure 1.**
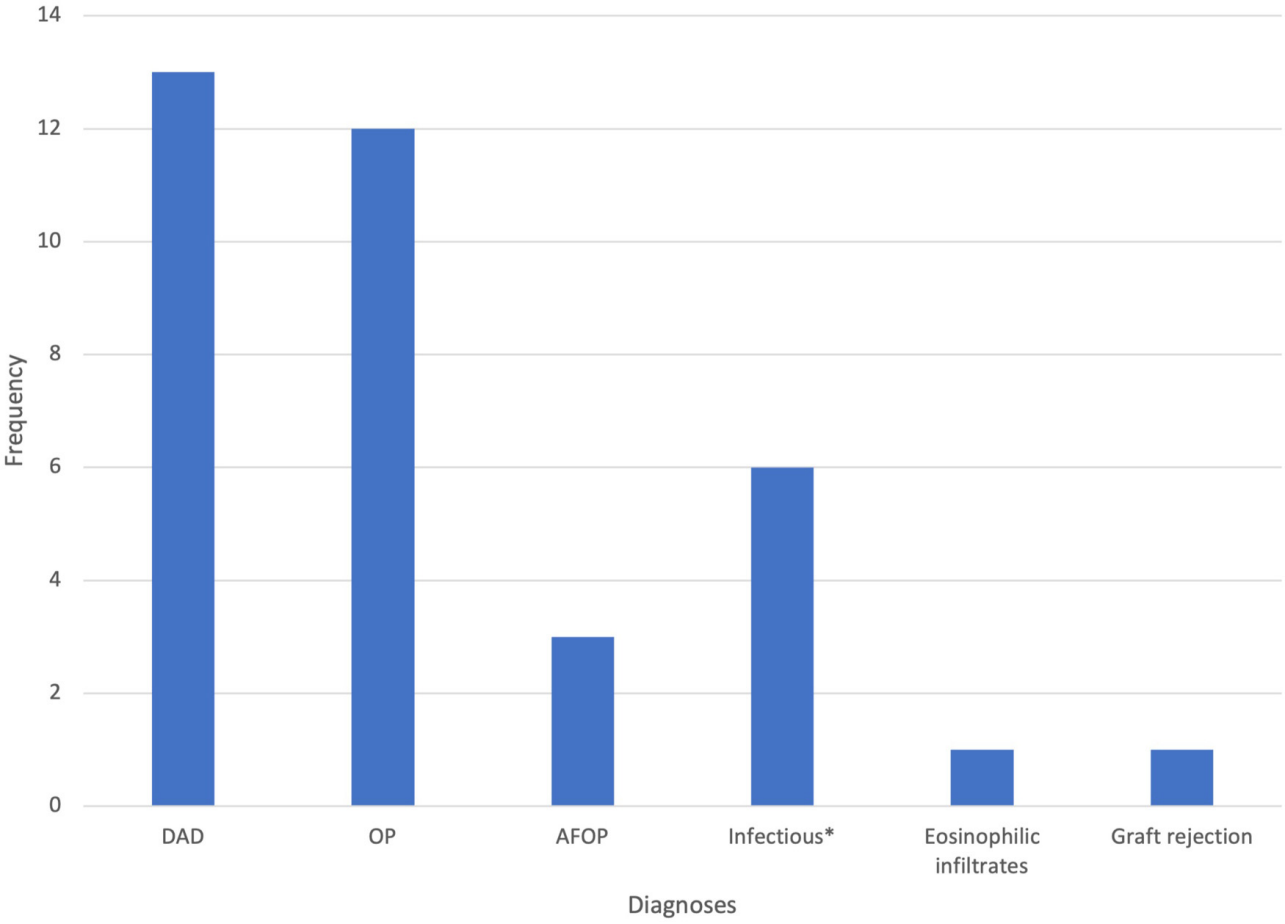
Histological diagnoses DAD, diffuse alveolar damage; OP, organizing pneumonia; AFOP, acute fibrinous organizing pneumonia; *infectious pathogens found in specimens: aspergillus, herpes simplex virus (HSV), cytomegalovirus (CMV), pneumocystis jiroveci, Stenotrophomonas maltophilia.

All biopsy specimens contained alveolar tissue. In 96% of cases (25 out of 26), histopathological diagnoses were made (see Table 3 and Figure 1). While histologic findings alone were only suggestive but not entirely diagnostic in several pathology reports, these cases were nonetheless considered diagnostic if the clinical evolution of the patient following biopsy-diagnosis-based management confirmed the suggested histologic diagnosis. One biopsy performed by TBLB showed possible organizing pneumonia (OP); however, the patient did not respond to an increase in immunosuppression, and thus the biopsy was deemed non-diagnostic. A total of 40 diagnoses were made with the remaining 25 biopsies. The most common results for all biopsy modalities were diffuse alveolar damage (DAD) (13 out of 40) and OP (12 out of 40). Eight out of 40 results were specific for an etiology: seven were infectious and one was due to graft rejection. Four of these diagnoses were made by SLB.

**Table 3.** Histopathological Diagnoses.

Histopathological diagnosis	TBLC n = 8	TBLB n = 10	SLB n = 8	Total n = 26
Diffuse alveolar damage (n, %)	3 (27)	5 (38)	5 (19)	13 (33)
Organizing pneumonia (n, %)	3 (27)	4 (31)	6 (22)	12 (30)
Acute fibrinous and organizing pneumonia (n, %)	2 (18)	2 (15)	0	4 (10)
Non-specific interstitial pneumonia (n, %)	0	0	1 (4)	1 (3)
Eosinophilic infiltrates (n, %)	1 (9)	0	0	1 (3)
Infection^a^ (n, %)	2 (18)	1 (8)	4 (15)	7 (18)
Graft rejection^b^ (n, %)	0	1 (8)	0	1 (3)
Total	11	13	27	40
No histological diagnosis^c^	0	1	0	1

DAD, diffuse alveolar damage; SLB, surgical lung biopsies; TBLB, transbronchial forceps lung biopsies; TBLC, transbronchial lung cryobiopsies.

^a^ Infectious pathogens found in specimens: aspergillus, herpes simplex virus (HSV), cytomegalovirus (CMV), Pneumocystis jiroveci, Stenotrophomonas maltophilia

^b^ Acute cellular rejection A2B0C0.

^c^ Histology showed possible organizing pneumonia, but since the patient did not respond to an increase in immunosuppression the biopsy was deemed non-diagnostic.

**Figure 2.**
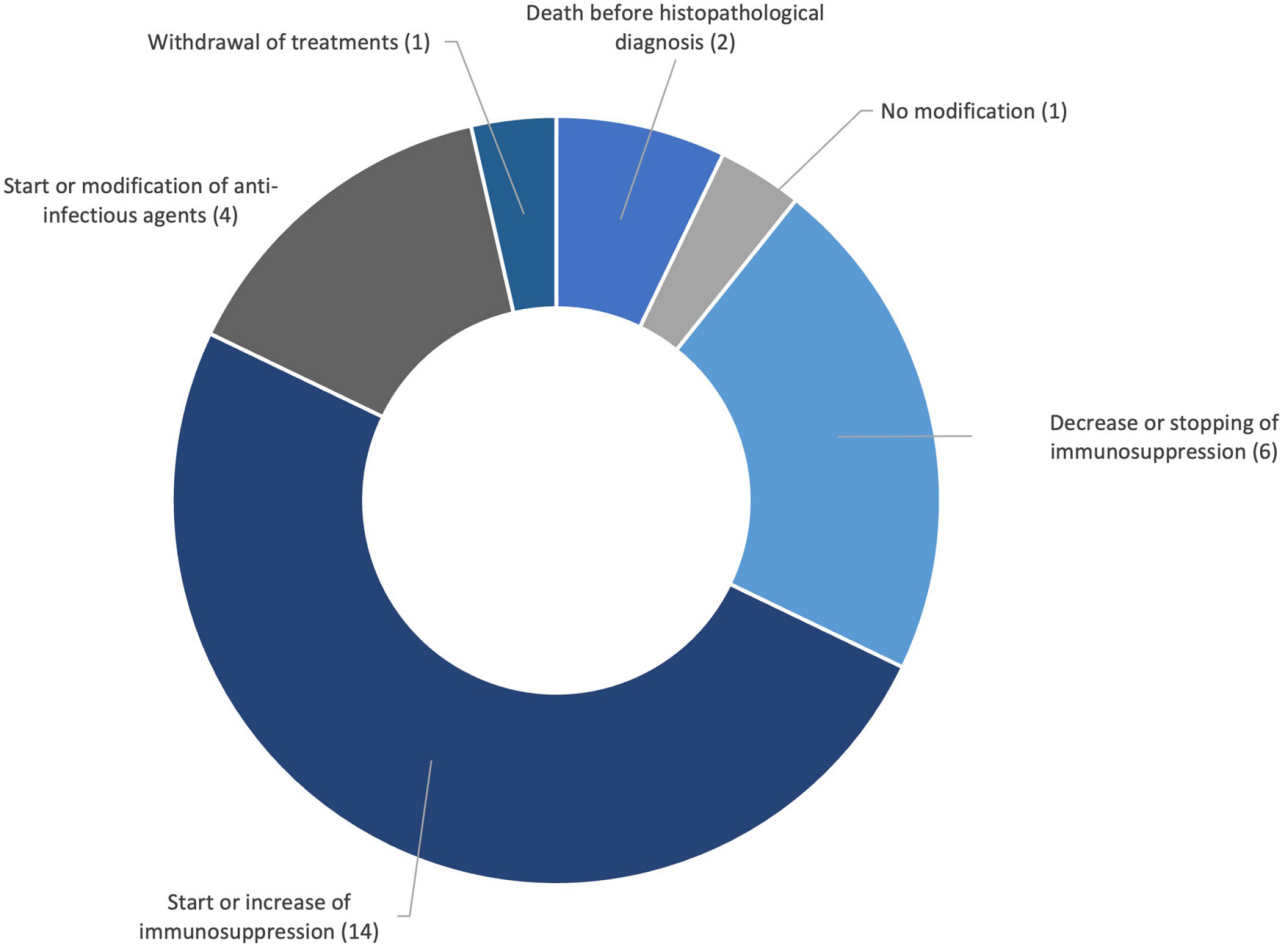
Treatment modifications.

Biopsy results led to treatment modifications in 23 patients (see Table 4 and Figure 2). The most common modification, in accordance with histological findings, was the initiation or escalation of immunosuppressants (14 out of 25). Antimicrobials were started or changed based on the histological diagnosis in four patients. One patient who was already treated for Pseudomonas and Aspergillus found in her BAL had a biopsy specimen culture that was positive for Stenotrophomonas maltophilia and was started on tigecycline. However, she remained in refractory shock despite treatments and was subsequently switched to palliative care. Of note, the Stenotrophomonas was only found on the culture of the lung tissue and not in the BAL. Two patients in whom infectious pathogens were found in the lung biopsy specimens died before the results were available. One patient who had refractory neoplasia, worsening AHRF, and active *Pneumocystis jirovecii* pneumonia on biopsy specimens despite receiving second-line antibiotic treatment was deemed to have a poor prognosis and was therefore switched to palliative care. One patient who had DAD on a TBLB specimen while already receiving empirical antibiotics and corticosteroids did not have treatment modification.

**Table 4.** Treatment Modifications After Biopsy Results.

Modification of treatment	TBLC n = 8	TBLB n = 10	SLB n = 8	Total n = 26
Start of immunosuppressors (n, %)	3 (38)	4 (40)	3 (38)	10 (40)
Increase of immunosuppressors (n, %)	1 (13)	1 (10)	2 (25)	4 (16)
Stopping of immunosuppressors (n, %)	1 (13)	0	1 (13)	2 (8)
Decrease of immunosuppressors (n, %)	0	4 (40)	0	4 (16)
Start or modification of antimicrobials (n, %)	1 (13)	0	3 (38)	4 (16)
Withdrawal of treatments (n, %)	1 (13)	0	0	1 (4)
Total modifications (n)	7	9	9	25
Death before biopsy results (n, %)	1 (13)	0	1 (13)	2 (8)
No modification (n, %)	0	1 (10)	0	1 (4)

SLB, surgical lung biopsies; TBLB, transbronchial forceps lung biopsies; TBLC, transbronchial lung cryobiopsies.

## Discussion

We observed high proportions of patients who experienced at least one adverse event following biopsy using any of the three methods: TBLC, TBLB, and SLB. Although our study had limited statistical power, we found that TBLC had the highest unadjusted and confounded numbers of total and severe complications, while TBLB had the lowest. Patients undergoing TBLC also had worse baseline disease characteristics, including higher SOFA scores and lower PaO2/FiO2 ratio, shorter duration of mechanical ventilation prior to biopsy, and shorter survival time compared to the other two groups. All three biopsy methods had high diagnostic yields and contributed to patient management. ICU and hospital mortality were high for all three groups.

There is limited evidence comparing the complication rates of different lung biopsy methods in AHRF of unknown etiology. However, a previous study found similar results, with a more critically ill TBLC patient group experiencing more adverse events.^5^ The higher complication rates in TBLC may be due to selection bias, as these patients may be referred for TBLC rather than SLB due to their poor prognosis and inability to undergo transportation to the operating room, single-lung ventilation, and post-operative risks. TBLC may also result in more complications compared to TBLB due to the larger specimens they extract, which may cause larger pneumothoraxes and more bleeding. The performance of TBLC without fluoroscopy and systematic bronchial blockers in our center may also contribute to these higher rates of adverse events.

Diagnostic and treatment modification rates were comparable to other studies for TBLC (72%-100%) and SLB (78%-80%), while rates for TBLB were higher than previously observed (41%-58%).^5,14–16^ This higher diagnostic yield may be explained by the larger numbers of specimens taken during our TBLB procedures compared to some prior studies. Performing culture on biopsy specimens may add a diagnostic value in revealing infectious pathogens that were not found by the pre-biopsy ancillary testing, as was the case for one of our patients.

Despite high diagnostic yields and contribution to patient management, ICU and hospital mortality rates were high and greater than mortality rates generally observed in ARDS patients,^26^ likely due to the severity of our patients’ baseline illnesses and comorbidities. Of note, TBLB patients, who were mainly immunocompromised lung transplant recipients, had the highest hospital mortality despite having lower comparative numbers of post-biopsy complications. This finding is similar to a previous work describing SLB in patients with severe ARDS and organ system failures, which also showed high mortality despite high diagnostic yields.^27^The high prevalence of DAD in our patients also reflects their critical condition, as DAD has been associated with lower PaO2/FiO2 ratios, higher plateau pressures, and higher mortality in ARDS.^8,28^ In addition, since we defined a positive diagnostic yield by the presence of alveolar tissue on histology, all of our biopsies were considered diagnostic, but most did not lead to specific etiologies of ARHF. The small sizes of the biopsy specimens may also have missed heterogeneously distributed and potentially treatable causes, especially with the transbronchial methods. In previous works, higher numbers of etiologically specific diagnoses were obtained with post-biopsy multidisciplinary discussions, which contributed to more treatment modifications^5,21^ and was associated with better survival.^5^ In our patients, multidisciplinary discussions and diagnostic tests for AHRF were not consistently conducted, which may have led to incomplete investigations, treatment plans, and higher mortality.

Our study has several limitations. First, it is a retrospective, single-center study with small sample sizes that were restricted by the number of biopsies performed from the moment of introduction of TBLC in our centre's ICU (early 2018) to the start of this study (early 2022). There are also selection biases for patients undergoing TBLC and TBLB, as the former group is generally referred due to their poor prognosis, and the latter group consists mainly of lung transplant recipients who had lung biopsy performed by their treating pulmonologist to rule out graft rejection. In addition, the TBLC and TBLB procedures at our center may differ from those at other centers in terms of the use of fluoroscopy and bronchial blockers. Bleeding risks of TBLC in critically ill patients were only seldomly reported when our center started to use TBLC in 2018. Furthermore, in-ICU mobile fluoroscopy is not available in our ICU. Since patients in our TBLC group were in general more severely ill, with uncertain bleeding risks related to TBLC, we opted not to transport them out of the ICU, estimating the risk of moving the patients higher than the risk of conducting the procedure in the ICU without moving the patients with trained staff readily available. It remains unclear whether the prophylactic use of bronchial blocker would reduce the risk of complications or increase the overall risk of other complications associated with routine bronchial blocker use. We thus did not use bronchial blockers prophylactically in our patients. Comparison with other studies may therefore be difficult due to the small sample sizes and heterogeneity of patient populations, biopsy techniques, reported diagnostic processes, and complications. Despite these limitations, our study provides valuable insights into the risks and benefits of lung biopsies in AHRF. Mortality rates remained high in this critically ill patient population, and further research is needed to identify which AHRF patients may benefit from lung biopsies and to determine the best biopsy modality.

## Supplemental Material

sj-docx-1-jic-10.1177_08850666241247145 - Supplemental material for Transbronchial Lung Cryobiopsies, Transbronchial Forceps Lung Biopsies, and Surgical Lung Biopsies in Mechanically Ventilated Patients with Acute Hypoxemic Respiratory Failure: A Retrospective Cohort Study Supplemental material, sj-docx-1-jic-10.1177_08850666241247145 for Transbronchial Lung Cryobiopsies, Transbronchial Forceps Lung Biopsies, and Surgical Lung Biopsies in Mechanically Ventilated Patients with Acute Hypoxemic Respiratory Failure: A Retrospective Cohort Study by Qi Li, Dominique Lafrance, Moishe Liberman, Charles Leduc, Emmanuel Charbonney, Polina Titova, Hélène Manganas and Michaël Chassé in Journal of Intensive Care Medicine
