# Supplementary material for: Transbronchial Lung Cryobiopsies, Transbronchial Forceps Lung Biopsies, and Surgical Lung Biopsies in Mechanically Ventilated Patients with Acute Hypoxemic Respiratory Failure: A Retrospective Cohort Study
Source: J Intensive Care Med. 2024 Apr 22;39(10):985–93. doi: 10.1177/08850666241247145 (PMC11528934; doi:10.1177/08850666241247145)
Supplement: sj-docx-1-jic-10.1177_08850666241247145 - Supplemental material for Transbronchial Lung Cryobiopsies, Transbronchial Forceps Lung Biopsies, and Surgical Lung Biopsies in Mechanically Ventilated Patients with Acute Hypoxemic Respiratory Failure: A Retrospective Cohort Study [file sj-docx-1-jic-10.1177_08850666241247145.docx]

# Supplement tables

**Supplement Table 1: Pre-biopsy clinical findings**

| Clinical findings | TBLC  n=8 | TBLB  n=10 | SLB  n=8 | Total  n=26 |
| --- | --- | --- | --- | --- |
| SOFA scores (mean)  Total  Respiratory  Cardiovascular  Coagulation  Glasgow Coma Scale  Bilirubin  Renal | 12.0  3.0  3.3  0.4  3.8  0.1  1.5 | 9.6  2.1  2.3  0.4  3.0  0.3  1.5 | 9.5  2.6  0.5  1.0  3.1  0.1  2.1 | 10.3  2.5  2.0  0.6  3.3  0.2  1.7 |
| Ventilation settings (mean)  Days under mechanical ventilation  FiO2 (%)  PEEP (cm H_2_O)  Tidal volume (ml)  Respiratory rate per minute  Peak pressure (cm H_2_O)  Inhaled pulmonary vasodilators (n, %)  ECMO (n, %)  Arterial blood gas values (mean)*  pH  PaO2 (mmHg)  PaO2/FiO2 | 7  69  9  333  28  33  2  1  7.38  82  139 | 15  42  8  355  26  30  1  0  7.36  94  239 | 10  55  10  366  23  28  1  1  7.34  91  205 | 11  54  9  351  26  30  4 (15)  2 (8)  7.36  89  198 |
| Empirical treatments (n, %)  Antibacterial, antiviral or antifungal  Corticosteroids | 8  4 | 10  4 | 7  5 | 25 (96)  13 (50) |

**Supplement Table 2: Pre-biopsy tests and medication management** (n, %)

| **Investigations and medications** | **All patients**  n=26 | **TBLC**  n=8 | **TBLB**  n=10 | **SLB**  n=8 |
| --- | --- | --- | --- | --- |
| BAL^a^ | 23 (88) | 8 (100) | 8 (80) | 7 (88) |
| BAL cell count^b^ | 9 (35) | 5 (63) | 1 (10) | 3 (38) |
| BAL cytology^b^ | 9 (35) | 3 (38) | 2 (20) | 3 (38) |
| Legionella urinary antigen^b^ | 14 (54) | 3 (38) | 4 (40) | 7 (88) |
| Histoplasma antigen^b^ | 4 (15) | 2 (25) | 4 (40) | 1 (13) |
| HIV | 15 (58) | 4 (50) | 1 (10) | 4 (50) |
| ANCA^c §^ | 9 (69) ^§^ | 6 (75) | 10 (100) | 6 (75) |
| Chest CT Scan^b^ | 23 (88) | 8 (100) | 8 (80) | 7 (88) |
| Transthoracic echocardiogram^c^ | 14 (54) | 3 (38) | 5 (50) | 6 (75) |
| Coagulation studies  *Ordered and normal^a^*  *Ordered and abnormal*^a^  *Not ordered* | 21 (81)  5 (19)  0 | 7 (88)  1 (13)  0 | 8 (80)  2 (20)  0 | 6 (75)  2 (25)  0 |
| No aspirin or on hold < 7 days prior to the biopsy | 24 (92) | 8 (100) | 9 (90) | 7 (88) |
| Prophylactic heparin on hold on the morning of the biopsy | 20 (77) | 7 (88) | 9 (90) | 4 (50) |
| No therapeutic anticoagulation < 48 hours prior to the biopsy | 23 (88) | 1 (13) | 2 (20) | 0 |

*BAL: bronchoalveolar lavage*

^a^*ordered < 24h prior to the biopsy*

^b^*ordered < 7 days prior to the biopsy*

^c^*ordered < 1 month prior to the biopsy*

*^§^exclusion of lung transplant and COVID patients*
